# Napsin A as a marker of clear cell ovarian carcinoma

**DOI:** 10.1186/1471-2407-13-524

**Published:** 2013-11-05

**Authors:** Ingiridur Skirnisdottir, Kathrine Bjersand, Helena Åkerud, Tomas Seidal

**Affiliations:** 1 Department of Women’s and Children’s Health, Uppsala University, SE-751 85 Uppsala, Sweden; 2 Department of Pathology, Halmstad Medical Center Hospital, Halmstad, Sweden

**Keywords:** Age, BMI, Capsule rupture, CCC, Concomitant p21p53, NAPA, Napsin A, Ovarian cancer, ROC curves

## Abstract

**Background:**

Clear cell carcinomas are aggressive tumors with a distinct biologic behaviour. In a genome-wide screening for genes involved in chemo-resistance, NAPA was over-expressed in cisplatin-resistant cells. The NAPA (protein) Napsin A was described to promote resistance to cisplatin by degradation of the tumor suppressor p53.

**Methods:**

Totally 131 patients were included in this study all in FIGO-stages I-II; 16 were clear cell tumors which were compared with 40 Type I tumors and 75 type II tumors according to the markers Napsin A, p21, p53 and p27 and some clinical features. For detection of the markers tissue microarrays and immunohistochemistry were used.

**Results:**

Positivity for Napsin A was detected in 12 (80%) out of the 15 clear cell tumors available for analysis compared with 3 (4%) out of the Type I and II tumors in one group (p < 0.001). Differences in p21 status, p53 status, and p21 + p53- status were striking when clear cell tumors were compared with Type I, Type II, and Type I and II tumors in one group, respectively. The p21 + p53-status was associated to positive staining of Napsin A (p = 0.0015) and clear cell morphology (p = 0.0003). In two separate multivariate logistic regression analyses with Napsin A as endpoint both clear cell carcinoma with OR = 153 (95% C.I. 21–1107); (p < 001) and p21 + p53- status with OR = 5.36 (95% C.I. 1.6-17.5); (p = 0.005) were independent predictive factors. ROC curves showed that AUC for Napsin A alone was 0.882, for p21 + p53- it was 0.720 and for p21 + p53-Napsin A + AUC was 0.795. Patients with clear cell tumors had lower (p = 0.013) BMI than Type I patients and were younger (p = 0.046) at diagnosis than Type II patients. Clear cell tumors had a higher frequency (p = 0.039) of capsule rupture at surgery than Type I and II tumors.

**Conclusions:**

Positivity of Napsin A in an epithelial ovarian tumor might strengthen the morphological diagnosis of clear cell ovarian carcinoma in the process of differential diagnosis between clear cell ovarian tumors and other histological subtypes.

## Background

Epithelial ovarian cancer (EOC) is the main cause of death among women with gynecologic malignancies [1]. At present, post-surgical therapy is mainly dependent upon tumor stage and grade rather than histological subtype [2]. On the basis of a series of morphologic and molecular genetic studies various types of ovarian cancer can be classified into two groups designated type I and type II [3,4]. Clear cell carcinoma (CCC), which constitutes 5-6% of ovarian malignancies exhibit morphologic, molecular, and clinical features that do not entirely resemble either Type I or Type II tumors and unlike, other Type I tumors, clear cell carcinoma CCC is high grade at presentation [4,5].

Consequently, most authorities in the field recommend that ovarian clear cell carcinomas should be automatically classified as grade 3 [2,3]. This is also in agreement with recent findings from Zannoni et al. [4], where they show that clear cell ovarian carcinoma should be studied separately, but still in comparison with the groups of Type I and Type II tumors. In a study from Chan et al. [6] it was concluded, that women with clear cell ovarian carcinoma are likely to be younger at diagnosis. Clear cell carcinoma usually presents as a pelvic mass with higher frequency of capsule rupture than other subtypes and it is known since before that this subtype is associated with endometriosis [7,8]. Clear cell ovarian tumors were furthermore, in a large clinical trial, found to present mostly as FIGO-stages I/II at diagnosis [9].

Many studies have found that clear cell carcinoma has a distinct aggressive biologic behaviour with poor response to platinum-based therapy compared to other subtypes of epithelial ovarian cancer [6]. Ovarian clear cell carcinoma may be more closely related to other clear cell tumors than other subtypes of ovarian cancer [10]. Cisplatin act mainly by inducing apoptosis in cancer cells and it has been shown in mouse studies as well as in the clinics that intact wild-type p53 is required for efficient execution of apoptosis. In clear cell carcinoma wild-type p53 is mostly present and mutations are uncommon [10,11].

Recently, it was shown in genome-wide screening for genes involved in chemo-resistance, that NAPA consistently was over-expressed in cisplatin-resistant cells [12]. The gene NAPA has been detected on chromosome 19q 13.33 and the corresponding protein Napsin A is an aspartic peptidase [13]. It was found to represent an anti-apoptotic protein that promotes resistance to cisplatin by degradation of the tumor suppressor p53, which is regulator of the cell cycle and co-works with cyclin kinase inhibitors as p21 and p27 [14-16]. The p21 gene is a primary mediator of p53-induced cell cycle arrest [17]. Napsin A is known to be present in primary lung adenocarcinomas as well as renal cell carcinoma (papillary and clear cell subtypes). As Napsin A is usually absent in the neoplastic cells in squamous carcinoma (pulmonary and non-pulmonary) it has been used as a diagnostic tool to distinguish between these two types of tumors [18].

## Methods

### Study population

A total of 140 consecutive patients with FIGO-stage I-II epithelial ovarian cancer, who underwent primary surgery and post-surgical chemotherapy in the Uppsala-Örebro Medical Region during at the 5-year period from January 1, 2000 to December 31, 2004, were entered into this study. All samples were collected with the patient’s informed consent and were in compliance with the Helsinki Declaration [19] and used in accordance with the Swedish Biobank Legislation and Ethical Review Act (approval by Uppsala Ethical Review Board, decision ref.UPS-03-477).

In total, 131 patients were included in this study and there were 131 available tumors for analysis of p53 and p27, 129 tumors for analysis of p21, and 124 tumors available for analysis of Napsin A (lower numbers because of technical issues in the staining process).

The primary surgery was performed at nine different surgical gynecological departments and the staging procedure was done at the time of primary surgery. Modified surgical staging according to the EORTC surgical staging categories in early ovarian cancer [20] was undertaken in 34 (26%) out of the 131 cases, and in the remaining 77 (74%) patients surgical staging was regarded as minimal or inadequate according to the same guidelines. All patients had chemotherapy 4–6 weeks after primary surgery. In the total series 105 out of the 131 (80%) of patients received paclitaxel 175 mg/m^2^ and carboplatin (AUC = 5) at 3-week intervals usually in four courses. The remaining 26 patients were treated with single-drug carboplatin in 4–6 courses. No patients were lost from clinical follow-up and the mean follow-up time was 65 months (range 5–110 months). Survival was defined as date of confirmed histological diagnosis after primary surgery to date of recurrence, death or last visit.

### Ovarian tissue microarray and Immunohistochemistry

The specimens were obtained from the paraffin blocks containing the embedded tissue removed from the tumor at primary surgery and after staining with hemotoxylin and eosin they were classified and graded by a single pathologist. The tissue microarrays were constructed as described previously [21]. In brief, tumor tissues were embedded in paraffin and 5 μm sections stained with hematoxylin-eosin were obtained to select representative areas for biopsies. Core tissue biopsy specimens (diameter 0.6 mm) were taken from these areas of individual donor paraffin blocks and precisely arrayed into a new recipient paraffin block with a custom-built instrument. Tissue core specimens from 131 ovarian carcinomas were arranged in three recipient paraffin blocks. Two core biopsies were obtained from each specimen. The presence of tumor tissue on the arrayed samples was verified by hematoxylin-eosin-stained section by a pathologist.

Five μm thick sections were cut from each multi tissue block and were put on coated slides and dried overnight at 37°C. The sections were pre-treated by heath-induced epitope retrieval in target- retrieval solution (Dako), pH = 6 or EDTA buffer pH = 9, for 7 + 7 minutes in microwave oven (99°C). Blocking with peroxidase was performed for 5 minutes. The slides were counterstained with hematoxylin for 2 minutes. The following monoclonal primary antibodies were used: NCL-L Napsin A (dilution 1:400, mouse monoclonal antibody, Novocastra, Newcastle, UK), DO-7, directed against p53 protein (dilution 1:1000; Dako, Glostrup, Denmark), p21 protein (dilution 1:50; Dako, Glostrup, Denmark) and NCL-p27 (dilution 1:40; Vision Biosystems Novocastra, Newcastle, UK). The immunostainings were performed in an Autostainer automated machine (Dako) using REAL Envision detection system (Dako). The work of tissue-microarray construction was undertaken at the Department of Pathology, the University Hospital MAS Malmö, Sweden, but the immunohistochemical analyses as well as the interpretation were performed at the Department of Pathology, Halmstad Medical Central Hospital, Sweden.

### Interpretation

The immunohistochemical (IHC) stains were interpreted by two of the authors (IS and TS). At the time for evaluation, no information was available on the specific diagnosis and prognosis for the individual cases. A semi-quantitative analysis [22] was used and the stainings were graded as negative, +, ++, and +++ for Napsin A, p21, p53 and p27. All markers were dichotomized into negative and positive (+, ++, +++) cases [23].

**Figure 1:**
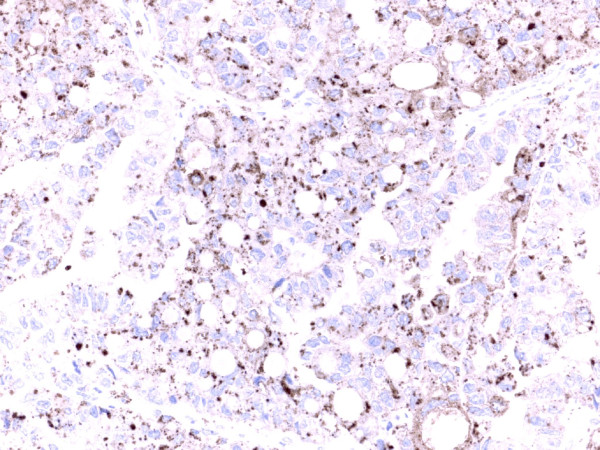
Section of ovarian clear cell carcinoma demonstrating strong (+++) Napsin A positivity as granular cytoplasmic staining in the tumor cells.

The staining for Napsin A in the tumor cells was considered to be positive (Figure 1) if there was a distinct granular staining in the cytoplasm and no background staining could be detected. In Additional files IHC pictures from sections of ovarian clear cell carcinoma are shown. Thus, an IHC picture in an Additional file 1 was demonstrating weak (+) Napsin A positivity, an IHC picture in an Additional file 2 was demonstrating moderate (++) Napsin A positivity and an IHC picture in an Additional file 3 was demonstrating strong (+++) Napsin A positivity. In an Additional file 4 Napsin A negativity in a section of ovarian clear cell carcinoma was demonstrated.

The staining for p21 and p53 was considered to be positive when there was a strong and granular staining of the nuclei of the majority of tumor cells. Finally, the staining for p27 was considered to be positive when strong and granular staining of the nuclei and cytoplasm of the tumor cells was found.

### Statistical analysis

The Pearson’s Chi-square test was used for testing proportional differences in univariate analyses. The survival curves were generated by using the Kaplan-Meier technique and differences between these curves were tested by the log-rank test. All tests were two-sided and the level of statistical significance was p < 0.05. By means of Receiver operating characteristic (ROC) curves Area under the curve (AUC) was determined for Napsin A, p21 + p53- and p21 + p53-Napsin A+. For multivariate analyses the logistic regression model was used with positivity of Napsin A as the end point. The Statistica11.0 (StatSoft™) statistical package for personal computers was used for the analyses.

## Results

### Background characteristics

Patients’ characteristics, e.g. age, performance status (according to WHO), FIGO-stage, histology, and FIGO-grade are demonstrated in Table 1. The study population included 40 type I tumors (30.5%), 75 type II tumors (57.3%), and 16 clear cell carcinomas (12.2%) shown in Table 2. Primary cure was achieved in all 131 patients (100%). The total number of recurrences in the complete series was 34 out of 131 (26%), and 22 of these patients (67.0%) died due to their disease. Five patients (15%) with recurrent disease died due to intercurrent disease and 7 (18%) patients were still alive at the last follow-up.

**Table 1:** Patients’characteristics (N=131)

**Age (median)**	**59.0**	**(Range 25-84)**
WHO performance status		(%)
0	37	(28.2)
1	66	(50.4)
2	21	(16.0)
3	6	(4.6)
FIGO-stage		
IA	39	(29.8)
IB	6	(4.6)
IC	66	(50.4)
II	20	(15.3)
Histopathology		
Serous	51	(38.9)
Mucinous	20	(15.3)
Endometrioid	42	(32.1)
Clear cell*	16	(12.2)
Anaplastic	2	(1.5)
FIGO-grade		
Grade 1	31	(23.7)
Grade 2	45	(34.4)
Grade 3*	55	(41.9)

Clear cell* (all clear cell tumors were graded as grade 3 tumors).

**Table 2:** Type I tumors and type II tumors according to combination of histological subtype and FIGO-grade

**Types of ovarian tumors (after histopathology and tumor grade)**	**N**	**(%)**
**Type I tumors**	40	**(**30.5)
Low-grade (G1) serous	14	
Mucinous (G1+G2+G3)	20	
Low-grade (G1) endometrioid	6	
**Type II tumors**	75	(57.3)
High-grade (G2+G3) serous	37	
High-grade (G2+G3) endometrioid	36	
Anaplastic	2	
**Clear cell tumors**	16	(12.2)

Clear cell tumors were all graded as G3 tumors.

In the complete series, recurrent disease was significantly associated with FIGO sub-stages (p = 0.0005), FIGO-grade (p = 0.030), adequate surgical staging (p = 0.033) and residual disease (p = 0.001). However, histopathology, capsule rupture at surgery and ascites at primary surgery were not associated with recurrent disease. In the complete series the 5-year disease-free survival rate was 68%, the disease-specific survival rate 76%, and the overall survival rate 71%.

### Immunohistochemistry

*Staining of Napsin A* was confined to the cytoplasm of the tumor cells. Positivity for Napsin A was observed in 16 (13%) out of the 124 tumors available for interpretation of this marker. In 15 out of the 16 clear cell tumors results from IHC for Napsin A were available. Positivity for Napsin A was detected in 12 (80%) out of the 15 clear cell tumors (Table 3). The difference was highly significant compared to Type I tumors, type II tumors and Type I and II tumors in one group, respectively. Status of Napsin A was associated to p21p53 status. Thus, concomitant positivity for p21 and negativity for p53 staining was detected in 10 out of the 15 tumors with positivity for Napsin A (67%) compared with 28 out of 107 (26%) tumors with negativity for Napsin A (p = 0.0015). The Napsin A status was not related to FIGO-stage or recurrent disease. In survival analysis there was no differences in 5 year disease-free survival after the Napsin A status (Log-rank = 28.017; p = 0.822).

**Table 3:** Status of protein expression in tumors of the Napsin A, p21, p53, p21 + p53-/other in one group and p27 versus clear cell tumors compared to Type I tumors, Type II tumors and Type I and II tumors

	**N (%)**	**N (%)**	**N (%)**	**N (%)**	**N (%)**	**N (%)**
	**16 (29)**	**40 (61)**	**16 (18)**	**75 (82)**	**16 (12)**	**115 (88)**
	**Clear cell**	**Type I**	**Clear cell**	**Type II**	**Clear cell**	**Other (Type I+II)**
	**Tumors**	**Tumors**	**Tumors**	**Tumors**	**Tumors**	**Tumors**
**Positivity**						
NapsinA+	12 (80)	1 (3)	12 (80)	3 (4)	12 (80)	4 (4)
NapsinA-	3 (20)	34 (97)	3 (20)	71 (96)	3 (20)	105 (96)
	p = 0.0000		p = 0.0000		p = 0.0000	(chi-2)
p21+	11 (69)	11 (28)	11 (69)	24 (32)	11 (69)	35 (31)
p21-	5 (31)	28 (72)	5 (31)	50 (68)	5 (31)	78 (69)
	p = 0.005		p = 0.007		p = 0.003	(chi-2)
p53+	0 (00)	11 (28)	0 (00)	22 (29)	0 (00)	33 (29)
p53-	16 (100)	29 (72)	16 (100)	53 (71)	16 (100)	82 (71)
	p = 0.019		p = 0.012		p = 0.013	(chi-2)
p21+p53-	11 (69)	7 (18)	11 (69)	21 (28)	11 (69)	28 (25)
Other#	5 (31)	32 (82)	5 (31)	53 (72)	5 (31)	85 (75)
	p = 0.0003		p = 0.002		p = 0.0003	(chi-2)
p27+	11 (69)	12 (30)	11 (69)	52 (69)	11 (69)	64 (56)
p27-	5 (31)	28 (70)	5 (31)	23 (31)	5 (31)	51 (44)
	p = 0.007		p = 0.963		p = 0.321	(chi-2)
Other# (p21+p53+, p21-p53+, p21-p53-)						

### Staining of p53, p21 and p27

In previous studies [24,25] including the total series of patients (N = 131), results from IHC for p21, p27 and p53 have been presented. Status of protein expression in tumors of p21 (N = 129) and p27 and p53 (N = 131) was analysed according to clinico-pathological features (age, histopathological subtype, FIGO-grade, FIGO-stages, and recurrent disease) and survival. The distribution of four subgroups was analysed based on the p53 status, p21 status and p27 status of tumors according to the same variables. Furthermore, in a previous study [25] the complete series of 129 patients was split into two subgroups according to concomitant p21- and p53+ of the tumors compared with other in one group (p21 + p53+, p21 + p53-, p21-p53-) and the distribution of the subgroups were analysed according to the same features as before.

### Concomitant staining of p21 and p53

In the present study, the p21 p53 status was split into two subgroups according to concomitant p21 + p53- in one group (N = 39) compared with other combinations (p21 + p53+, p21-p53+, p21-p53-) in a second group (N = 90) (Table 3). Among clear cell tumors 11 (69%) out of the 16 carcinomas were belonging to the subgroup of concomitant positivity for p21 and negativity for p53 compared with the remaining five (31%) clear tumors in the study where other combinations of p21 p53 status were presented.

This was different from Type I tumors (p = 0.0003), type II tumors (p = 0.002) and Type I and II tumors in one group (p = 0.0003) (Table 3). Furthermore, only one (5%) out of the 19 mucinous tumors had concomitant staining for p21+ p53- (p = 0.0007) (data not shown). Status of concomitant p21+ and p53- in tumors compared with other combinations (p21 + p53+, p21-p53+, p21-p53-) in one group was not associated to FIGO-stage (p = 0.853) or recurrent disease (p = 0.062). In survival analysis there were no differences in 5 year survival between the group of patients with p21 + p53- in tumors compared with the group of patients, whose tumors had other combinations of p21p53 status (Log-rank = 9.552; p = 0.341).

### Clear cell versus Type I ovarian carcinoma

The mean age of patients with clear cell carcinomas (54.7 years) did not significantly differ from that in Type I group (57.2 years) (Table 4). However, the group of patients with clear cell carcinomas had significantly (p = 0.013) lower BMI compared to the Type I group. No difference according to the FIGO sub-stages or recurrent disease between the two groups of patients could be detected. However, capsule rupture occurred more frequently (p = 0.039) in clear cell tumors compared with Type I tumors. There was no difference in disease –free survival between the group of patients with clear cell tumors compared with the group of patients with Type I tumors (Log-rank = 29.690; p = 0.623).

**Table 4:** Clinical features of clear cell tumors compared to Type I tumors, Type II tumors and Type I and II tumors in one group

	**N (%)**	**N (%)**	**N (%)**	**N (%)**	**N (%)**	**N (%)**
	**16 (29)**	**40 (61)**	**16 (18)**	**75 (82)**	**16 (12)**	**115 (88)**
	**Clear cell**	**Type I**	**Clear cell**	**Type II**	**Clear cell**	**Other (Type I+II)**
	**Tumors**	**Tumors**	**Tumors**	**Tumors**	**Tumors**	**Tumors**
Age	54.7	57.2	54.7	60.2	54.7	59.1
	p = 0.388		p = 0.046		p = 0.100	(t-test)
BMI ≤ 25	11 (73)	11 (36)	11 (73)	44 (61)	11 (73)	58 (52)
BMI > 25	4 (27)	25 (64)	4 (27)	28 (39)	4 (27)	53 (48)
	p = 0.013		p = 0.372		p = 0.123	(chi-2)
STAGE						
IA-B	3 (19)	20 (50)	3 (19)	22 (29)	3 (19)	42 (19)
IC	10 (62)	16 (40)	10 (62)	40 (54)	10 (62)	56 (49)
II	3 (19)	4 (10)	3 (19)	13 (17)	3 (19)	17 (15)
	p = 0.097		p = 0.669		p = 0.341	(chi-2)
Capsule						
Rupture*						
Yes	10 (63)	13 (33)	10 (63)	39(34)	10 (63)	26 (35)
No	6 (37)	27 (67)	6 (37)	76 (66)	6 (37)	49 (65)
	p = 0.039		p = 0.027		p = 0.039	(chi-2)
Recurrence						
Without	12 (75)	31 (77)	12 (75)	54 (69)	12 (75)	85 (74)
With	4 (25)	9 (23)	4 (25)	21 (31)	4 (25)	30 (26)
	p = 0.841		p = 0.807		p = 0.926	(chi-2)

* Capsule of the tumor is perforated before or at primary surgery.

Differences in Napsin A status were highly significant between the two groups (Table 3). Thus, 12 out of the 15 (80%) clear cell carcinomas stained positively for Napsin A compared with only one out of the 35 (3%) Type I tumors. Differences in the p21 status, p53 status, p21 + p53- versus other combinations in one group and p27 status were also striking between the two groups (p = 0.005), (p = 0.019), (p = 0.003) and (p = 0.007), respectively. In summary clear cell carcinomas usually stained positively for Napsin A, p21 and p27, but conversely p53 was absent alone or in combination with positive staining for p21.

### Clear cell versus Type II ovarian carcinoma

The patients with clear cell carcinomas was significantly (54.7 years) younger than patients in the Type II group (60.2 years) (p = 0.046). However, no differences were found according to BMI divided in two groups, FIGO sub-stages or recurrent disease (Table 4) between the two groups of patients, whereas capsular rupture occurred more frequently (p = 0.027) in clear cell tumors compared with Type II tumors. There was no difference in disease –free survival between the group of patients with clear cell tumors compared with the group of patients with Type II tumors (Log-rank = 29.690; p = 0.623).

Likewise, the differences in immunohistochemical profile for all markers, with exception of p27 were striking between the two groups of clear cell and Type II tumors (Table 3) in the same manner as before in comparison with Type I tumors.

### Clear cell versus Type I and II ovarian carcinoma in one group

The mean age of patients with clear cell carcinomas did not significantly differ from that in Type I and II in one group. No differences were found according to BMI in two groups, FIGO sub-stages or recurrent disease between the two groups of patients with clear cell tumors and patients belonging to Type I and II in one group. However, capsular rupture occurred more frequently (p = 0.039) in the group of patients with clear cell carcinomas. In survival analysis, disease free survival was not different (Log-rank = 32.977; p = 0.835) between the two subgroups of patients.

Clear cell tumors could be considered biologically different from other histological subtypes of epithelial ovarian cancer. Thus, differences in the immunohistochemical profil for Napsin A and the apoptosis regulators p21, p53 and concomitant p21 and p53 between the groups of clear cell carcinomas and other histological subtypes of tumors in one group (Type I and Type II) could be detected in the present study.

### Multivariate analysis

In multivariate logistic regression analysis (Table 5) with positivity of Napsin A in ovarian tumors as endpoint, clear cell tumors was the only independent predictive factor (OR = 153, 95% C.I. 21–1107, p < 0.001) in analysis together with age, FIGO sub-stages (I / II) and tumor grade (G1/G2 + G3). The variables “clear cell tumors” and “p21 + p53- status” was not introduced together in the multivariate models because of a strong correlation (p < 0.001) between them. In a separate multivariate analysis (together with the same variables as before) (Table 5) the p21 + p53- status versus other combinations of p21p53 status in one group was an independent predictive factor (OR 5.36, 95% C.I. 1.65-17.48, p = 0.005).

**Table 5:** Predictive factors for positivity of Napsin A (logistic regression analysis)

**Variable**	**OR**	**95% C.I.**	**P value**
Age	0.974	0.917–1.036	0.410
Stage (I vs II)	3.197	0.276–37.054	0.347
Grade	0.942	0.089–9.913	0.962
Clear cell*	152.661	21.042–1107.543	p<0.001
**Variable**	**OR**	**95% C.I.**	**P value**
Age	0.998	0.953–1.045	0.941
Stage (I vs II)	0.640	0.120–3.392	0.597
Grade	4.071	0.473–35.038	0.196
P21+p53-**	5.362	1.645–17.476	0.005

Grade# (G1 vs G2 + G3).

Clear cell* (Clear cell vs Type I and Type II tumors in one group (serous, mucinous, endometrioid and anaplastic)).

p21+p53-** (p21+p53-) vs (p21+p53+, p21-p53+, p21-p53-).

**Figure 2:**
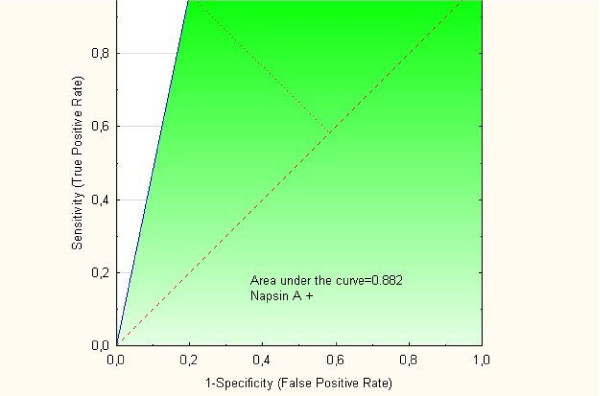
ROC curve for “Napsin A phenotype”.

**Figure 3:**
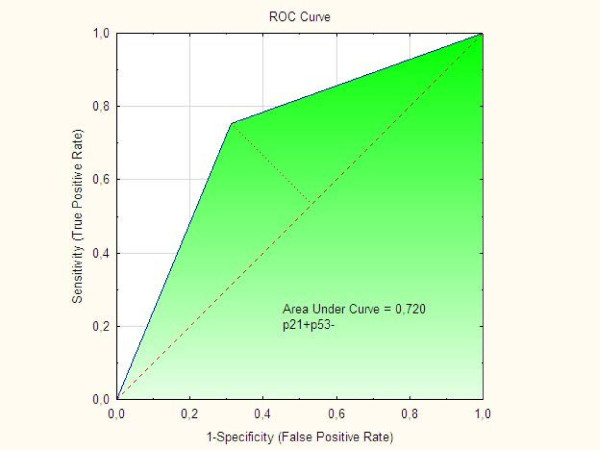
ROC curve for “p21 + p53- phenotype”.

**Figure 4:**
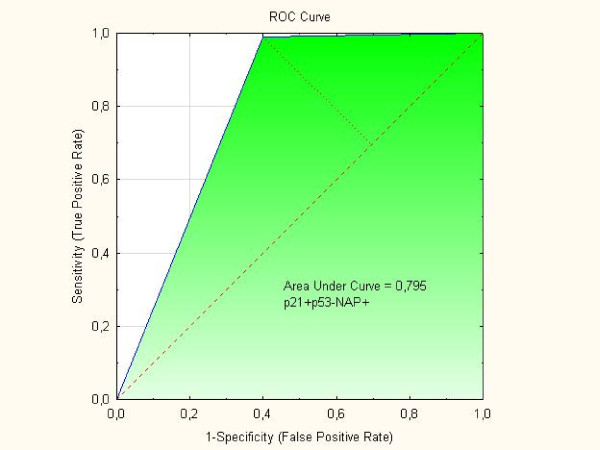
ROC curve for concomitant “p21 + p53-Napsin A + phenotype.

The predictive value of the markers Napsin A, p21 + p53- and p21 + p53-Napsin A + were evaluated by ROC curves. As demonstrated in Figure 2 the AUC for Napsin A alone was 0.882 and in Figure 3 the AUC for p21 + p53- was 0.720. However, as shown in Figure 4, the AUC for concomitant p21 + p53- and Napsin A+ decreased to 0.795 compared with the AUC for Napsin A alone.

## Discussion

In the present study it was confirmed that ovarian clear cell carcinomas exhibit clinico-pathological and immuno-histochemical features that is different from classical Type I and Type II tumors. The immunohistochemical profil based on staining for Napsin A and the apoptosis regulators p21, p53 and concomitant p21 and p53 were unique in the group of clear cell carcinomas compared to other histological subtypes of tumors (Type I and Type II).

Our findings related to the clinico-pathological differences are supported by earlier studies [4,7,10] but results about the immunohistochemical profile for Napsin A is new.

Clear cell carcinomas resembles Type I tumors based on relative genetic stability and frequent presentation in stage I, but on the other hand, clear cell carcinoma is high grade at diagnosis. Furthermore, wild-type p53 is mostly present and mutations are uncommon in clear cell tumors contrary to Type II tumors, which are genetically unstable and have a high frequency of p53 mutations [10,11]. Ovarian clear cell carcinomas constitute a heterogeneous disease at the genomic level despite having similar histological features. Previous data from Tan et al. [11] has suggested that the pattern of genome-wide copy number aberrations may predict clinical outcome.

Based on our results positivity for Napsin A seems to be more frequently detected in clear cell carcinomas than in other histological subtypes. The results are novel but in agreement with results from Kandalaft et al. [26] who has shown that Napsin A is highly expressed in 12 out of 12 (100%) ovarian clear cell tumors. We show further that concomitant positivity for p21 and negativity for p53 was strongly associated to positive staining for Napsin A and clear cell tumors, respectively in the present study. Some difficulties may arise in the morphological diagnosis between clear cell carcinomas and high grade serous or endometrioid ovarian carcinomas [10]. Therefore, concomitant positivity for p21 and negativity for p53 of a Napsin A positive ovarian tumor might strengthen the pathological diagnosis of clear cell ovarian carcinoma. Thus, our findings may facilitate the morphological diagnosis of clear cell ovarian carcinoma when there are difficulties in the pathological distinction between clear cell carcinomas and high grade serous or endometrioid ovarian carcinomas [10].

No single marker has been reported to be useful alone to distinguish between high grade serous and clear cell ovarian carcinoma. In a newly presented study where seven different markers for distinction between high grade serous and clear cell ovarian carcinoma did though p53 in combination with p21 (and Cyclin E, ER, HNF-1b, WT1 and Ki-67) correctly classify 84% of tumors with high grade serous compared with clear cell ovarian carcinoma [27]. Furthermore, as p53 missense mutations and consequent overexpression (positivity) is infrequent in clear cell carcinomas, but common in high-grade serous carcinoma, positive staining for p53 could be a potential marker to distinguish between these two tumors [27]. In the present study none of the 16 clear cell carcinoma showed positive p53 staining and our findings are in agreement with others [28,29].

Differences in the immunohistochemical profile for Napsin A and the apoptosis regulators p21, p53 and concomitant p21 and p53 between the groups of clear cell carcinomas and other histological subtypes of tumors in one group (Type I and Type II) were detected in the present study, whereas differences in the p27 status were limited to comparison between clear cell tumors and Type I tumors.

Findings from ROC curves in the present study showed that the markers Napsin A, p21 + p53- or p21 + p53-Napsin A + all had predictive values which might indicate that they are potential diagnostic markers in the process of differential diagnosis between clear cell ovarian carcinomas and other histological subtypes. However, we found that the p21 + p53- phenotype did not add to the Napsin A phenotype along the process of differential diagnosis between clear cell ovarian tumors and histological subtypes.

In a study on ovarian A2780 cells, it was demonstrated that p27 up-regulation is linked directly to activation of the p21/p53 pathway by a DNA-damaging agent (a cisplatin analogue). Therefore, the p53/p21/p27 axis should become a new focus of attention in checkpoint response to DNA damage [30].

In the clinics it is not always obvious how to handle women with suspected clear cells carcinoma, which most probably is related to its heterogeneity. Ovarian cancer is usually treated by surgery followed by chemotherapy. Though in the group of women diagnosed with clear cells carcinoma defined by commonly used histopathology a platinum resistance often occurs [6]. Based on the results described in this study we propose that Napsin A could not only be used as a diagnostic marker of clear cells carcinoma, but also might Napsin A positivity of clear cell carcinoma predict about platinum sensitivity. Out of there the detection of Napsin A in clear cell carcinoma could help us to understand how it promotes resistance to cisplatin by degradation of the tumor suppressor p53 [12,14].

Similarities between clear cell carcinomas and Type I tumors, Type II tumors and Type I and Type II tumors in one group, respectively were limited to FIGO-stages and recurrent disease.

Patients with clear cell carcinomas were younger than the Type II tumor patients (55 vs. 60 years; mean age). In SEER data reported by Chan et al. [6], women with clear cell histology were younger than patients with serous cancers. The fraction of patients with BMI > 25 was significantly lower in women with clear cell carcinoma compared to woman with type I tumors. This is in line with previous studies that have reported an elevated risk for epithelial ovarian cancer in woman with higher BMI [31]. This risk seams to be limited to the histological subgroups of low-grade serous and low to intermediate grade endometroid [32].

In our material, clear cell carcinomas showed a significantly higher frequency of capsular rupture compared to type I tumors, Type II tumors and Type I/type II combined. Even these results are in line with earlier findings and likely to contribute to poor prognosis for clear cell carcinomas. In one study improvement was observed in the 5-year disease-free survival for patients in FIGO-stage I without tumor rupture during surgery [33]. Rupture (before and during surgery) was the second most powerful prognostic indicator of disease-free survival (after the degree of differentiation) in a multicenter study including 1545 patients with invasive epithelial ovarian cancer all in FIGO-stage I [34].

In the present study the 5-year-survival was not different for the subgroups of patients with clear cell ovarian carcinoma from survival for patients with Type I tumors, Type II tumors or Type I and II in one group. A number of studies [35] have reported that overall survival of women with clear cell ovarian carcinoma is higher than in high grade serous cancer patients in the low-stages (FIGO I-II), although the survival was worse among women with clear cell ovarian carcinoma in the high stages (FIGO III-IV). However, in one of the largest series to date including 1411 clear cell patients comparing 5-year disease-specific survival rate after adjusting for stage there was a significantly worse survival rate associated with clear cell histology compared with other histological subtypes [6].

NAPA was identified as one of nine cisplatin resistance genes in a genome-wide analysis of HeLa cells by using DNA microarrays [12]. The chemotherapeutic agent cisplatin is known to induce apoptosis in actively replicating cells. Mouse studies and clinical evidence suggest that wild p53 is required for efficient apoptosis in tumor cells indicating that intact p53 represents a critical target of chemotherapeutic drugs [36,37].

Epithelial- mesenchymal transition (EMT) is a multistep process, by which polarized epithelial cells lose epithelial adherence and become capable of free movement through the extracellular matrix. This process is often activated during cancer invasion and metastasis [38]. Recently, it has become clear that mutant p53 proteins after losing their transcriptional function can acquire new functions and drive cell migration, invasion and metastasis [39]. It was reported from one study [40] that wild type p53 can inhibit the focal adhesion kinase (FAK) promoter activity in vitro, but FAK is a critical regulator of adhesion, motility, metastasis and survival signalling. Cells without Napsin A appear susceptible to transition and one of the reasons might be low-level expression of Napsin A. In a further study [38] it was demonstrated in an *in vitro* EMT model that, Napsin A caused G(0)/(G1) arrest and inhibited the expression of FAK. Recent reports [7] involving large institutional cohorts compared low-stage to high-stage ovarian cancers (I/II vs. III/IV) showed that 57–81% of clear cell carcinoma were diagnosed at stage I/II. One reason of the low frequency of high-stage disease in clear cell ovarian cancer could theoretically be explained by the findings from our study of high frequency of Napsin A positivity and p53 negativity (intact wild type p53) which both might inhibit the process of EMT in clear cell carcinoma.

It has been suggested that Napsin A may have a therapeutic potential as a gene therapy candidate for tumor metastasis as increase in expression of Napsin A and may inhibit the epithelial- mesenchymal transition [41]. In the future the development of high-throughput gene expression and genomic microarray- based analytical platforms may answer many important questions about clear cell ovarian carcinomas [10]. Firstly, if clear cell ovarian carcinomas are more closely related in molecular terms to other clear cell tumors as renal cell carcinomas or endometriod clear cell carcinomas? Does the potential exist for crossover therapeutic targets to be developed for clear cell tumors from a variety of tissue type? Therapy by using the gene NAPA and the protein, Napsin A could be one option of many for treatment of clear cell ovarian tumors from of a variety of tissues in the future.

Some limitations of this work have to be noted. One limitation corresponds to the relative limited number of patients included in the study, which though is conducted in a geographically well defined region in Sweden. However, the frequency of 16 (12.2%) patients with clear cell ovarian carcinoma out 131 consecutive patients all with low-stages epithelial ovarian cancer is in line with the frequency (12.4%) in a previous study in the same region [28]. Furthermore, the non-serous (mucinous, endometrioid and clear cell) subtypes usually are detected in FIGO-stages I-II and therefore represent uncommon diseases which require large scale research trials for searching for subtype specific biomarkers [8]. Another limitation is related to the tissue microarray technology used in this study might also contribute to some limitations of the work. Only two 0.6 mm core biopsies were obtained from each specimen for analysis and there could be a risk of non- representative tissue collected for microarray. As ovarian carcinomas can be very heterogeneous, such specimens may not be representative of the tumor in some cases. Out of there, we used the method of semi-quantitative analysis [22] for the interpretation. Thus, all markers were dichotomized into negative and positive groups [23]. However, in recent years, it has been common practice to perform immunohistochemical studies on tissue microarray, where a large number of antibodies can been screened in a rapid and cost efficient manner [18].

## Conclusions

Differences in the immunohistochemical profil for Napsin A and the apoptosis regulators p21, p53 and concomitant p21 and p53 between the groups of clear cell carcinomas and other histological subtypes of tumors in one group (Type I and Type II) were found in the present study, whereas differences in the p27 status were limited to comparison between clear cell tumors and Type I tumors. According to the ROC curves we found that the markers Napsin A, p21 + p53- and p21 + p53-Napsin A + all had some predictive value as diagnostic markers for clear cell ovarian carcinoma. However, the p21 + p53- phenotype did not add to the Napsin A phenotype alone along the process of differential diagnosis between clear cell ovarian tumors and histological subtypes.

## Competing interests

The authors declare that they have no competing interests.

## Authors’ contributions

IS: Collection and characterization of patient material; statistical analysis. Formulation of research questions, supervision of data analysis and interpretation of results. HÅ: Interpretation of results and formulation of research questions, KB: Interpretation of results. TS: Interpretation of results and formulation of research questions. IS, KB, HÅ and TS wrote the paper. All authors read and approved the final manuscript.

## Pre-publication history

The pre-publication history for this paper can be accessed here:

http://www.biomedcentral.com/1471-2407/13/524/prepub

## Supplementary Material

Additional file 1 **IHC pictures from section of ovarian clear cell carcinoma demonstrating.** Weak (+) Napsin A positivity. Click here for file

Additional file 2 **IHC pictures from section of ovarian clear cell carcinoma demonstrating.** Moderate (++) Napsin A positivity. Click here for file

Additional file 3 **IHC pictures from section of ovarian clear cell carcinoma demonstrating.** Strong (+++) Napsin A positivity. Click here for file

Additional file 4 **IHC pictures from section of ovarian clear cell carcinoma demonstrating.** Napsin A negativity in a section of ovarian clear cell carcinoma. Click here for file
