# Supplementary figures and images for: Napsin A as a marker of clear cell ovarian carcinoma
Source: BMC Cancer. 2013 Nov 5;13:524. doi: 10.1186/1471-2407-13-524 (PMC4228360; doi:10.1186/1471-2407-13-524)

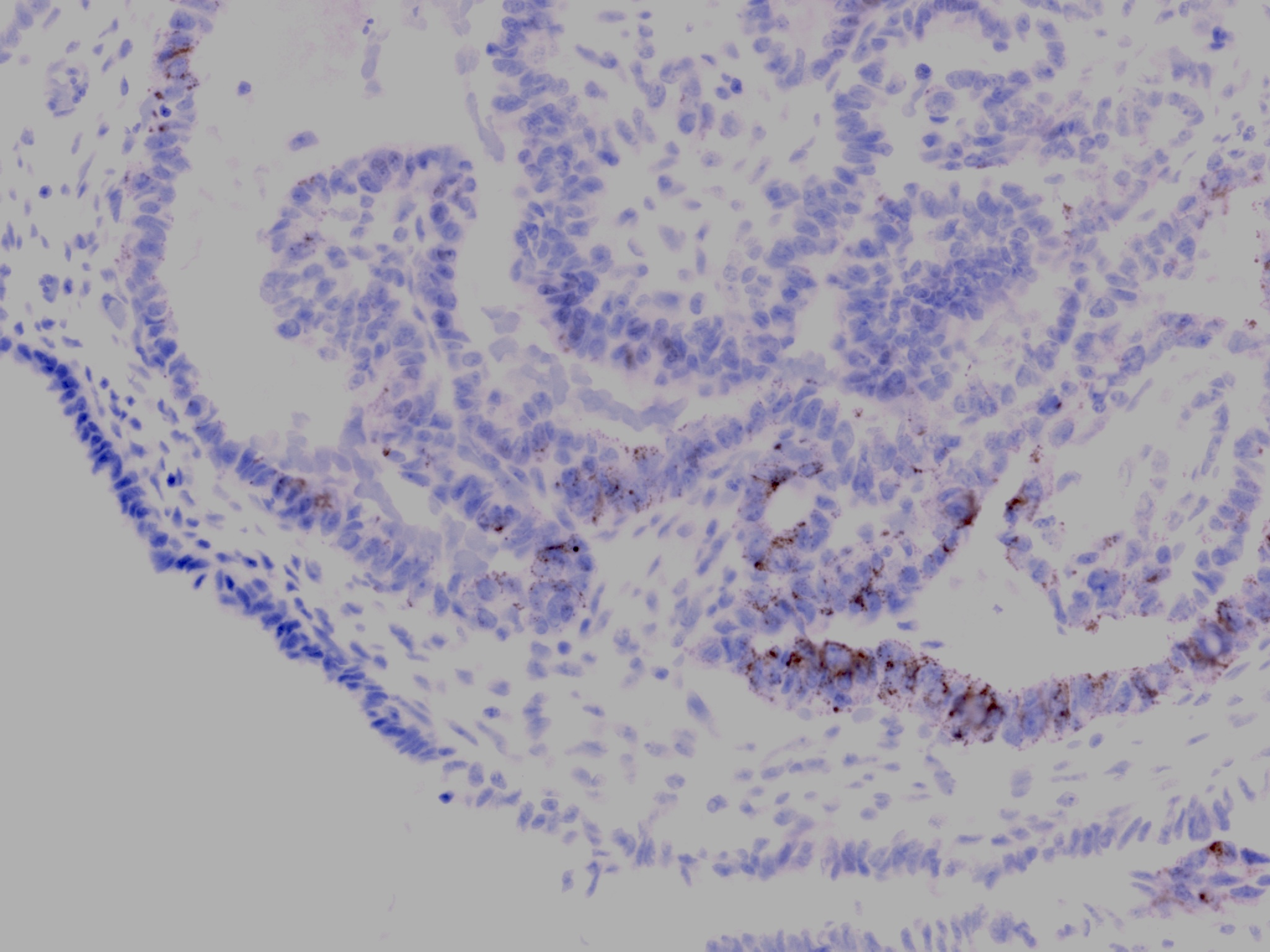

Supplement: Additional file 1 — IHC pictures from section of ovarian clear cell carcinoma demonstrating. Weak (+) Napsin A positivity. [file 1471-2407-13-524-S1.tiff]

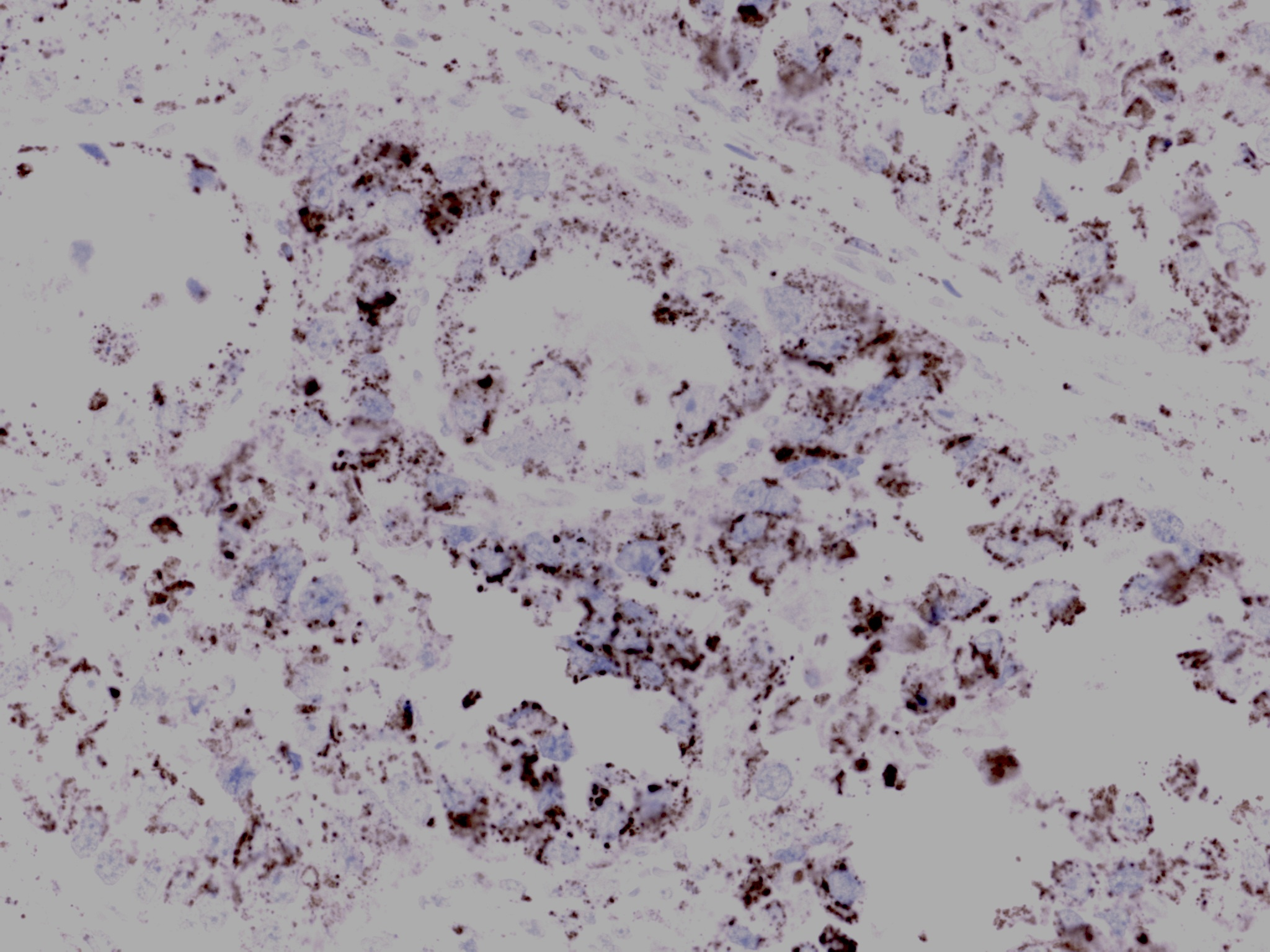

Supplement: Additional file 2 — IHC pictures from section of ovarian clear cell carcinoma demonstrating. Moderate (++) Napsin A positivity. [file 1471-2407-13-524-S2.tiff]

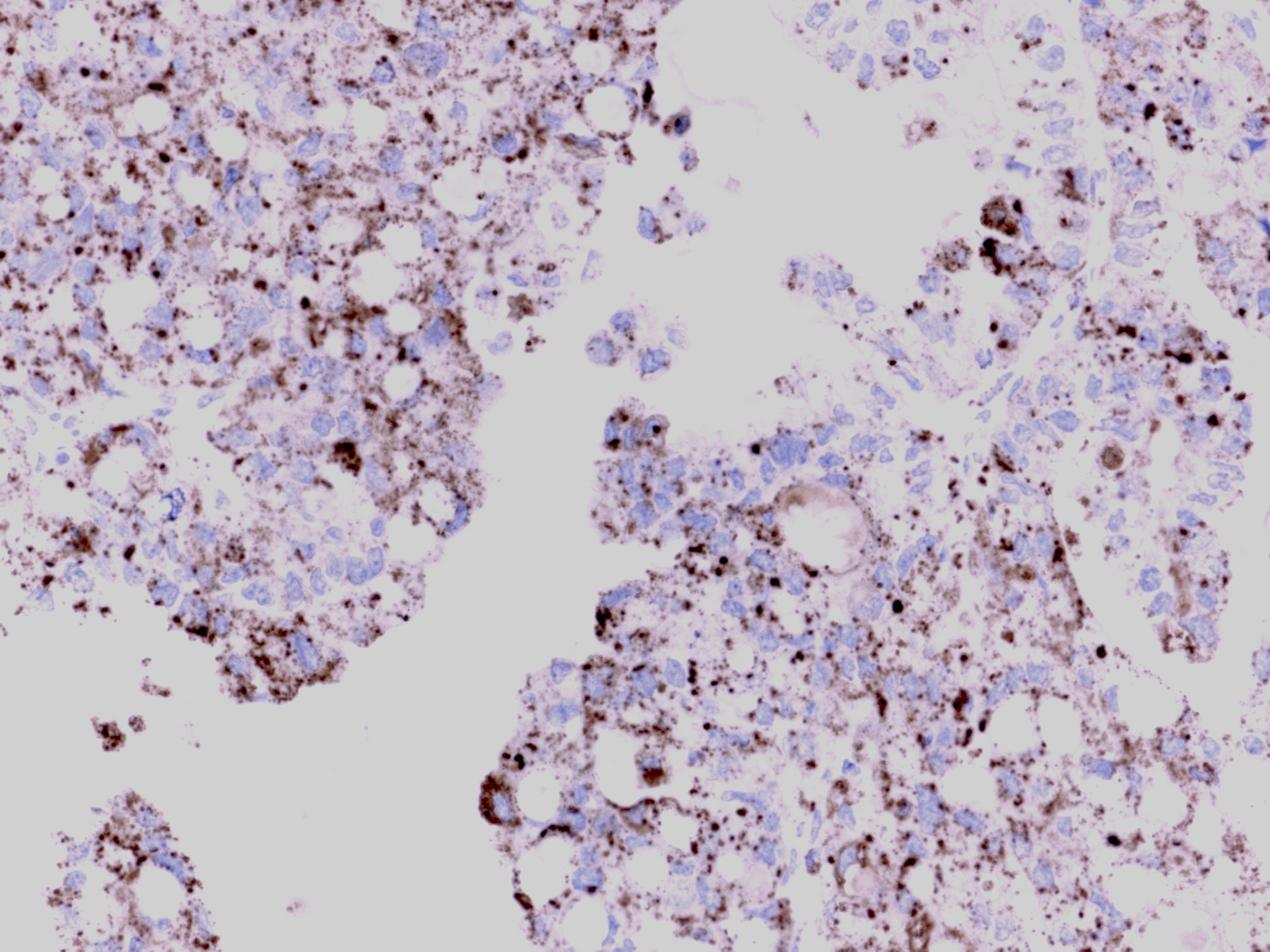

Supplement: Additional file 3 — IHC pictures from section of ovarian clear cell carcinoma demonstrating. Strong (+++) Napsin A positivity. [file 1471-2407-13-524-S3.tiff]

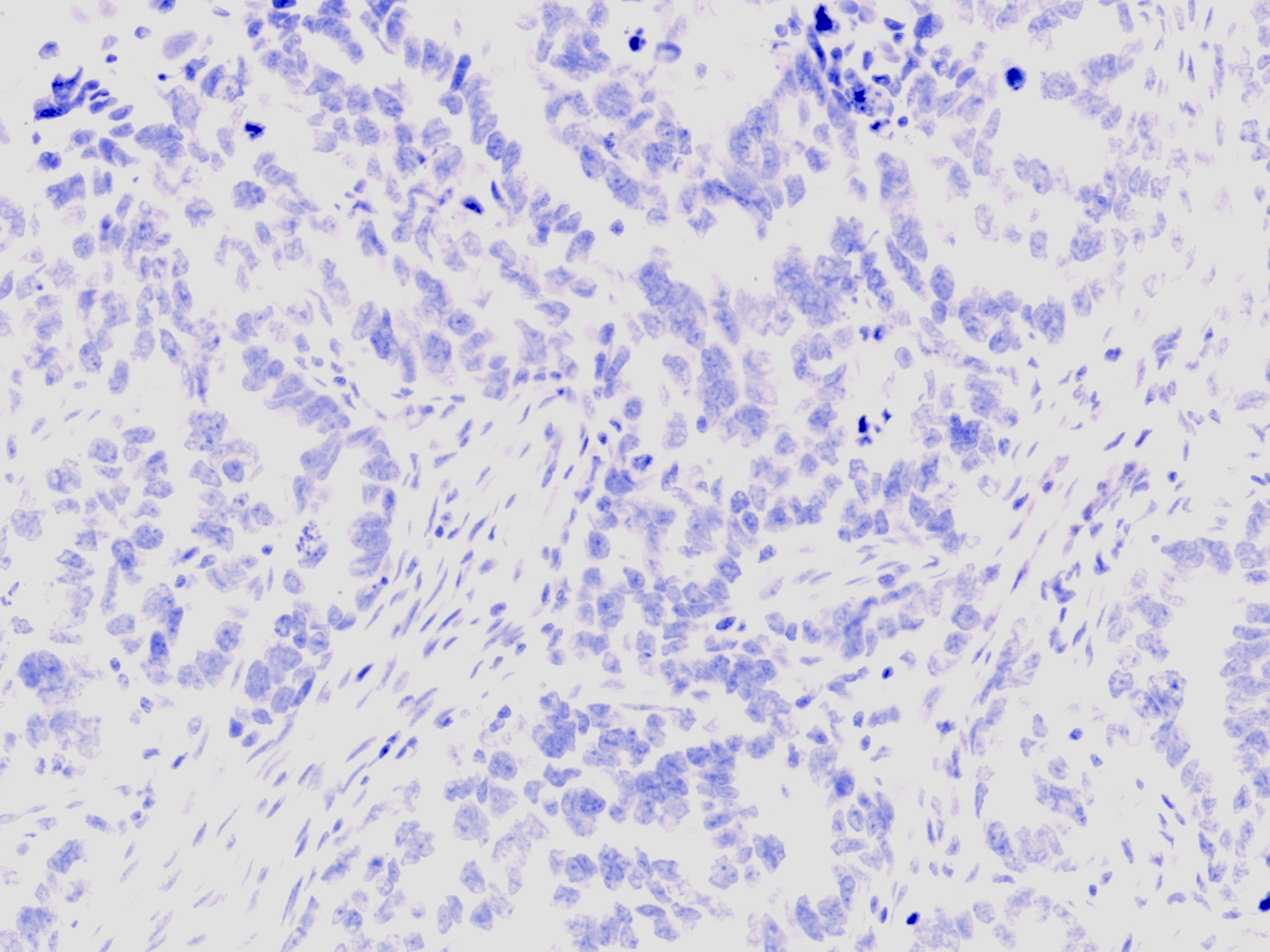

Supplement: Additional file 4 — IHC pictures from section of ovarian clear cell carcinoma demonstrating. Napsin A negativity in a section of ovarian clear cell carcinoma. [file 1471-2407-13-524-S4.tiff]
